# Evaluation of historical CMIP6 model simulations and future projections of temperature over the Pan-Third Pole region

**DOI:** 10.1007/s11356-021-17474-7

**Published:** 2021-12-01

**Authors:** Xuewei Fan, Qingyun Duan, Chenwei Shen, Yi Wu, Chang Xing

**Affiliations:** 1 State Key Laboratory of Earth Surface Processes and Resource Ecology, Faculty of Geographical Science, Beijing Normal University, Beijing, 100875 China; 2 College of Hydrology and Water Resources, Hohai University, Nanjing, 210098 China

**Keywords:** CMIP6, Temperature, The Pan-Third Pole, Climate change

## Abstract

The Pan-Third Pole (PTP) region, which encompasses the Eurasian highlands and their surroundings, has experienced unprecedented, accelerated warming during the past decades. This study evaluates the performance of historical simulation runs of the Coupled Model Intercomparison Project (CMIP6) in capturing spatial patterns and temporal variations observed over the PTP region for mean and extreme temperatures. In addition, projected changes in temperatures under four Shared Socioeconomic Pathway (SSP) scenarios (SSP1‐2.6, SSP2‐4.5, SSP3-7.0, and SSP5‐8.5) are also reported. Four indices were used to characterize changes in temperature extremes: the annual maximum value of daily maximum temperature (TXx), the annual minimum value of daily minimum temperature (TNn), and indices for the percentage of warm days (TX90p) and warm nights (TN90p). Results indicate that most CMIP6 models generally capture the characteristics of the observed mean and extreme temperatures over the PTP region, but there still are slight cold biases in the Tibetan Plateau. Future changes of mean and extreme temperatures demonstrate that a strong increase will occur for the entire PTP region during the twenty-first century under all four SSP scenarios. Between 2015 and 2099, ensemble area-averaged annual mean temperatures are projected to increase by 1.24 °C/100 year, 3.28 °C/100 year, 5.57 °C/100 year, and 7.40 °C/100 year for the SSP1-2.6, SSP2-4.5, SSP3-7.0, and SSP5-8.5 scenarios, respectively. For TXx and TNn, the most intense warming is projected in Central Asia. The greatest number of projected TX90p and TN90p will occur in the Southeast Asia and Tibetan Plateau, respectively.

**Supplementary Information:**

The online version contains supplementary material available at 10.1007/s11356-021-17474-7.

## Introduction

Warming of the climate system is unequivocal, a fact that has drawn overwhelming attention from the public, governments, and academic communities in recent decades (Gou et al. 2021). The warming has exerted profound, worldwide impacts on human life (Cheng et al. 2018; Sun et al. 2019), agricultural production (Liu et al. 2019, 2021; Tigchelaar et al. 2018), land use (Seneviratne et al. 2018), and natural ecosystems (Gou et al. 2020; Zheng et al. 2021). Among the areas expected to be sensitive to this warming is the region known as the Third Pole, which includes the Tibetan Plateau and the mountains surrounding it. Extending westward and northward from the Third Pole, the Pan-Third Pole (PTP) region is the core region of the “The Belt and Road” initiative promoted by the Chinese government, which has been building a new platform for international cooperation among more than 70 countries in Asia, Africa, and Europe. It covers more than 20 million km^2^ and supports over 3 billion people with its resources. The PTP region is among the regions in the world most vulnerable to the impact of climate change, since it has the world’s highest elevations and hosts the largest mass of glaciers and snow cover outside the polar regions (Wang et al. 2020). Under global warming, the PTP region, and especially the Tibetan Plateau, has experienced rates of warming twice the global average over the last 50–60 years (Deng et al. 2017; Pepin et al. 2019; You et al. 2019). Furthermore, the projected warming of some areas of the PTP region will exceed 4 °C above pre-industrial levels by 2100, which far exceeds the 2 °C goal set by the Paris Agreement of the United Nations Framework Convention on Climate Change (Yao et al. 2017). The warming in the PTP region is causing Earth system changes characterized by intensive interactions among the processes of the atmosphere, hydrosphere, cryosphere, and biosphere, and is resulting in environmental threats such as glacier retreat, ice collapse, glacial lake expansion, and frequent glacier lake outburst flood (Miao et al. 2021; Yang et al. 2014; Yao et al. 2019). These changes may have impacts on the regional and global hydrologic cycle, thereby hindering socioeconomic development in countries along the routes of the Belt and Road Initiative. A deeper understanding of climate changes in the PTP region can inform science-based adaptation strategies to reduce climate risks.

Global climate models (GCMs) have become a major and vital tool for projecting future changes in climate; their reliability depends on their ability to reproduce historical and current climate features. To this end, the World Climate Research Programme has promoted a set of experiments known as the Coupled Model Intercomparison Project (CMIP) since the 1990s, which have delivered systematic and high-quality simulations for better understanding past climate changes and making projections and uncertainty estimates of the future (Annan and Hargreaves 2011; Meehl et al. 2000). Studies using simulations from the fifth phase of CMIP (CMIP5; Taylor et al. 2012) have advanced our understanding of regionally heterogeneous climate warming (Bannister et al. 2017; Ongoma et al. 2018; Sun et al. 2017, 2020), highlighting strong warming trends in the high latitudes of the Northern Hemisphere and moderate warming trends in the middle latitudes (Feng et al. 2014). Several studies have investigated the performance of CMIP models for the PTP region. For example, Dong et al. (2018) assessed the performance of CMIP5 historical simulations and projected future changes under three Representative Concentration Pathways (RCP2.6, RCP4.5, and RCP8.5) over critical Belt and Road regions based on data from 22 models. They found the most significant areas of warming are expected in Kazakhstan and the northern part of the south Belt and Road region. Kamworapan and Surussavadee (2019) evaluated the performances of forty CMIP5 models for simulating climatological temperature and precipitation for Southeast Asia and suggest the use of an ensemble they called 6-GCM-Ensemble for climate studies and projections. Jia et al. (2019) comprehensively assessed the performance of 33 CMIP5 models by an improved-score‐based and multiple‐criteria method and demonstrated that most models could capture the seasonal temperature patterns.

The sixth phase of CMIP (CMIP6; Eyring et al. 2016) is now in progress, and it features updates to parameterizations, the addition of new physical processes, and somewhat higher resolution compared to CMIP5 (Eyring et al. 2019). CMIP6 contains future scenario experiments named the Scenario Model Intercomparison Project (ScenarioMIP; O'Neill et al. 2016; O’Neill et al. 2020), which produce projections for a new set of emissions and land use scenarios based on the Shared Socioeconomic Pathways (SSPs; Riahi et al. 2017). The SSPs describe plausible future changes in societal aspects such as demographic, technological, economic, social, governance, and environmental factors (O’Neill et al. 2017). The CMIP6 models provide a new opportunity to examine the climate system and make regional projections of the climate under new scenarios, including for the PTP region.

Before assessing projections into the future, it is essential to evaluate the credibility of CMIP6 simulations with respect to observations on regional scales. Recent studies have already investigated the performance of CMIP6 in simulating regional historical climate and achieved positive results (Fan et al. 2020b; Grose et al. 2020; Li et al. 2021; Lovino et al. 2021; Srivastava et al. 2020). However, a comprehensive assessment relative to historical observations over the PTP region has not yet been performed, especially for looking at the climate extremes. Similarly, projected climate changes under the new SSP scenarios have not yet been reported. Motivated by the above issues, this study aims to (1) evaluate the performance of the CMIP6 models in simulating near-surface mean and extreme temperatures over the PTP region during the historical period and (2) assess future climate change in the PTP region based on CMIP6 model simulations for the twenty-first century.

The paper is organized as follows: the “Data and methods” section briefly describes the study area and introduces the data and methodology used in this study. The performance of CMIP6 models in reproducing the observed temperature over the PTP region is assessed in the “Evaluations of historical temperature simulations” section. The “Projected temperatures in the twenty-first century” section investigates the projected changes of temperature over the different subregions of PTP in the twenty-first century. Finally, the “Summary and conclusions” section presents the main conclusions.

## Data and methods

### Study area

**Fig. 1:**
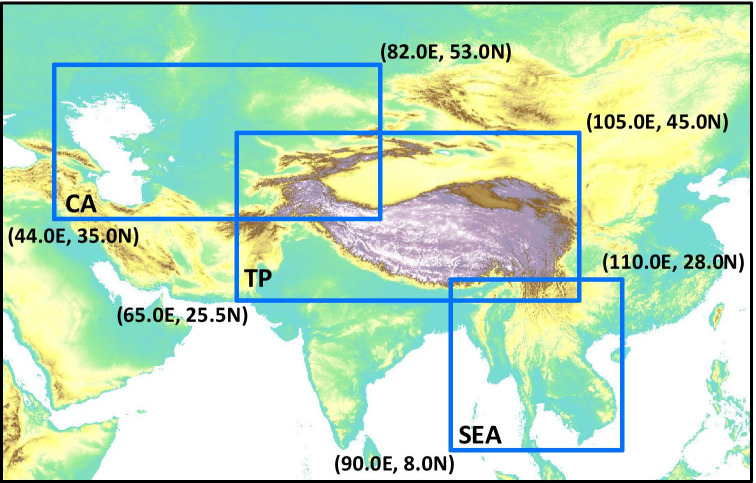
Location map of the study area. The blue rectangles represent the latitude and longitude ranges of the three subregions: Central Asia (CA), Tibetan Plateau (TP), and Southeast Asia (SEA)

The PTP region is located in one of the most fragile and rapidly developing regions of the Earth, around the Third Pole, which mainly includes the Tibetan Plateau and the northern intracontinental arid region of Asia, extending to the Caucasus Mountains in the west and the western Loess Plateau in the east (Yao et al. 2017). Figure 1 shows the areal extent considered in this study (35°E–125°E and 0°N–60°N) and its three subregions (Tibetan plateau (TP), Southeast Asia (SEA), and Central Asia (CA)). Home to three billion people, the PTP region hosts a substantial part of the “Silk Road Economic Belt” initiative proposed and advanced by China. The climate in the PTP region ranges from arid continental to humid tropical, primarily influenced by alternating predominance of the westerlies in winter and the Asian monsoon in summer. In turn, the PTP region exerts profound thermal and dynamic effects on atmospheric circulation, thus affecting the climate of Asia, the Northern Hemisphere, and beyond (Kang et al. 2019; Miao et al. 2019).

### CMIP6 datasets and observations

We obtained monthly simulations of near-surface temperatures and daily minimum and maximum near-surface temperatures from the CMIP6 database (https://esgf-node.llnl.gov/search/cmip6/) using the historical simulations (1850–2014) and future scenario simulations (2015–2099) from ScenarioMIP. ScenarioMIP recommends four Tier 1 simulation protocols, reflecting different SSPs that result in different radiative forcing magnitudes by 2100: SSP1‐2.6 (a low-forcing “sustainability” pathway; + 2.6 W/m^2^), SSP2‐4.5 (a medium-forcing “middle‐of‐the‐road” pathway; + 4.5 W/m^2^), SSP3‐7.0 (a medium‐ to high‐forcing pathway; + 7.0 W/m^2^), and SSP5‐8.5 (a high‐forcing pathway; + 8.5 W/m^2^). Table 1 provides the basic information of 16 GCMs that were involved to analyze the changes in annual mean temperature. Only the last 12 GCMs in Table 1 were used to analyze the changes in temperature extremes when considering the availability of daily data in historical experiment and four SSP scenarios.

**Table 1:** List of 16 CMIP6 models in this study and their spatial resolutions

Model name	Modeling center	Spatial resolution
BCC-CSM2-MR	Beijing Climate Center, China	320 × 160
CAMS-CSM1-0	Chinese Academy of Meteorological Sciences, China	320 × 160
CESM2-WACCM	National Center for Atmospheric Research, Climate and Global Dynamics Laboratory, United States	288 × 192
CESM2	National Center for Atmospheric Research, Climate and Global Dynamics Laboratory, United States	288 × 192
CNRM-CM6-1	National Centre for Meteorological Research, France	256 × 128
CNRM-ESM2-1	National Centre for Meteorological Research, France	256 × 128
CanESM5	Canadian Centre for Climate Modelling and Analysis, Environment and Climate Change Canada, Canada	128 × 64
EC-Earth3-Veg	EC-Earth Consortium, Europe	512 × 256
EC-Earth3	EC-Earth Consortium, Europe	512 × 256
FGOALS-g3	LASG, Institute of Atmospheric Physics, Chinese Academy of Sciences, China	180 × 80
GFDL-ESM4	National Oceanic and Atmospheric Administration, Geophysical Fluid Dynamics Laboratory, United States	288 × 180
IPSL-CM6A-LR	Institut Pierre Simon Laplace, France	144 × 143
MIROC-ES2L	JAMSTEC (Japan Agency for Marine-Earth Science and Technology), AORI (Atmosphere and Ocean Research Institute, The University of Tokyo), NIES (National Institute for Environmental Studies), and R-CCS (RIKEN Center for Computational Science), Japan	128 × 64
MIROC6	JAMSTEC, AORI, NIES and R-CCS, Japan	256 × 128
MRI-ESM2-0	Meteorological Research Institute, Japan	320 × 160
UKESM1-0-LL	Met Office Hadley Centre, United Kingdom	192 × 144

The reference mean temperature dataset used in this study is the gauge-based gridded Climatic Research Unit (CRU) TS v. 4.03 (Harris et al. 2020), which covers the period 1901–2018 with a 0.5° × 0.5° spatial resolution (available at https://crudata.uea.ac.uk/cru/data/hrg/cru_ts_4.03/). The dataset is derived by interpolating monthly climate anomalies from extensive networks of weather station observations, and it has been subject to extensive quality control measures. Numerous studies have utilized CRU datasets for temperature-related analysis and reported its capability in simulating temperature in various regions of globe (Ahmed et al. 2020; Fan et al. 2020a; Li et al. 2018; Osborn et al. 2021). All monthly simulations of CMIP6 models were bilinearly interpolated to a common 0.5° × 0.5° grid to keep the resolution consistent with CRU data.

The HadEX3 dataset (Dunn et al. 2020) is used to investigate observed changes in temperature extremes. This dataset is the recent global land-based climate extremes data developed by the World Meteorological Organization’s Expert Team on Climate Change Detection and Indices (ETCCDI), available on a 1.875° × 1.25° longitude-latitude grid for the period from 1901 to 2018 (https://www.metoffice.gov.uk/hadobs/hadex3).

### Methods

#### Calculation of extreme temperature indices

Four indices of temperature extremes (Table 2) from the ETCCDI were selected to assess historical and future changes related to daily maximum temperatures (TX) and daily minimum temperatures (TN) from 1951 to 2099. There are three hot indices (annual maximum value of TX, TXx; the percentage of warm days, TX90p; and the percentage of warm nights, TN90p) and one cold index (annual minimum value of TN, TNn), which together can characterize the intensity and frequency of temperatures extremes. These indices have been widely used to investigate the observed and projected changes in extreme temperatures (Li et al. 2019; Wehner 2020; Zhou et al. 2014). We calculated all indices at the models’ native resolution using the R package *climdex.pcic.ncdf* and regridded the outputs to a common 1° × 1° grid by bilinear interpolation to balance the resolution of HadEX3. The HadEX3 indices are also re-gridded to a 1° × 1° grid. When calculating the TX90p and TN90p indices, the same base period (1961–1990) from HadEX3 was applied to the CMIP6 models.

**Table 2:** Definitions of the extreme temperature indices used in this study

Label	Index name	Index definition	Units
TXx	Max TX	Annual maximum value of daily maximum temperature	°C
TNn	Min TN	Annual minimum value of daily minimum temperature	°C
TX90p	Warm days	Percentage of days when the daily maximum temperature is above the 90th percentile for the base period 1961–1990	%
TN90p	Warm nights	Percentage of days when the daily minimum temperature is above the 90th percentile for the base period 1961–1990	%

#### Model performance metric

Taylor diagrams were employed to quantify the degree of correspondence between model simulations and observations, taking into consideration mean temperature climatology patterns, which are shown by spatial correlation coefficients, root mean square error (RMSE), and the ratio of standard deviations (Taylor 2001). When the correlation coefficient and the standard deviation are close to 1 and the RMSE is close to 0, this is indicative of the best match between observation and model simulation. Additionally, we compared the average bias of the spatial pattern of the mean state of temperature between the observational data and the CMIP6 ensembles.

To evaluate the performance of the CMIP6 models in simulating temperature extremes for the PTP region, we construct a “portrait” diagram based on the relative model RMSE (RMSE′) of the climatology pattern for the period 1951–2014 (Sillmann et al. 2013). This diagram provides a concise graphical overview of the model performance for various temperature extreme indices. First, we calculated RMSEs for all individual models relative to the HadEX3 data. Then, the RMSEs for all models were collected to derive the relative model error for each model, RMSE′, defined as:

1 $${{RMSE}}^{{^{\prime}}}=\frac{{RMSE}-{{RMSE}}_{{median}}}{{{RMSE}}_{{median}}}$$

where RMSE_median_ is the median of RMSEs for individual models. Negative values of RMSE′ indicate that the corresponding model performs better than the majority (50%) of models.

## Evaluations of historical temperature simulations

### Evaluations of historical mean temperatures

**Fig. 2:**
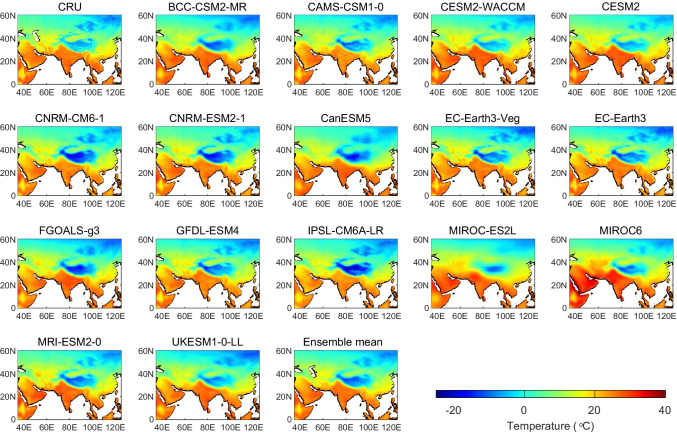
Spatial distributions of annual mean temperatures from 16 CMIP6 models, ensemble averages, and CRU observations over the PTP region for 1970–1999 average

The spatial distributions of annual mean temperatures from the 16 CMIP6 models and CRU observations over the PTP region are shown in Fig. 2. Generally, the observed annual mean temperature exhibits an increasing gradient from the north to south with the lowest temperatures occurring in the high-elevation TP. All the CMIP6 models reproduce the spatial distribution of annual temperature reasonably well and are able to capture the lowest temperatures in the TP. However, there are consistent cold biases in the TP and SEA for most models. Additionally, some models tend to overestimate the annual temperatures in South Asia, especially MIROC6 and MIROC-ES2L. The multi‐model mean results are notably similar to observations in most regions, and the biases are relatively smaller than those of most individual models.

**Fig. 3:**
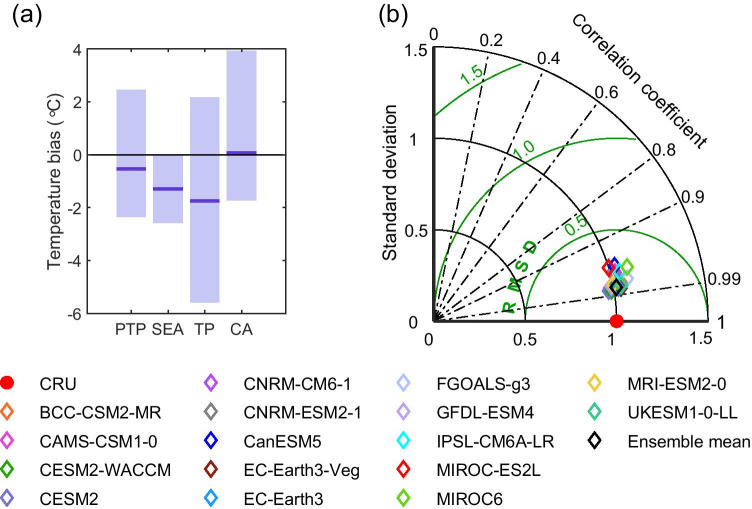
Area-averaged annual biases of temperature relative to the CRU observational dataset for each CMIP6 ensemble mean during 1970–1999 over the PTP region and its three subregions, (**a**) the light purple bars represent the maximum and minimum biases of the 16 GCMs, and the horizontal line in dark purple represents the bias of the multi-model mean. Taylor diagram displaying statistics of climatological annual mean temperature generated from 16 GCMs and CRU in the PTP region during 1970–1999, (**b**) the vertical axis indicates standard deviation ratios, the numbers along the arc are the spatial correlations, and the green circles centered at CRU measure RMSE difference relative to the standard deviation of the observations

Next, we use bias calculations and Taylor diagrams to quantitatively assess the performance of models in simulating the spatial pattern of annual mean temperature. The length of each bar in Fig. 3(a) shows the spread in regional average temperature biases simulated by the CMIP6 models relative to the CRU observations. We found that the overall biases of the PTP region vary between − 2.35 and 2.45 °C. Among the three subregions, the TP region exhibits the largest negative biases, while the CA region shows large positive biases. The bias of the CMIP6 multi-model mean is below 2 °C, which indicates smaller differences compared with the corresponding observed data than most models produce in simulating spatial patterns. Figure 3(b) depicts the Taylor diagram of the model simulations against observations, which summarizes the degree of correspondence between the observed and simulated fields. All 16 CMIP6 models are in line with observations, with correlation coefficients (dotted radial lines) all above 0.9. Also, most of the models exhibit a ratio of the standard deviations that is close to 1 and the centered pattern RMSE difference range was 0.17–0.30. This indicates that the CMIP6 models perform reasonably well in simulating the spatial distribution of annual mean temperatures over the PTP region. With respect to the statistical parameters in the Taylor diagram, CESM2-WACCM, CESM2, EC-Earth3, and EC-Earth3-Veg exhibit superior performance compared with other models. However, the CMIP6 ensemble average outperforms many of the individual models, as indicated by its closer distance to the reference point on the Taylor diagram.

**Fig. 4:**
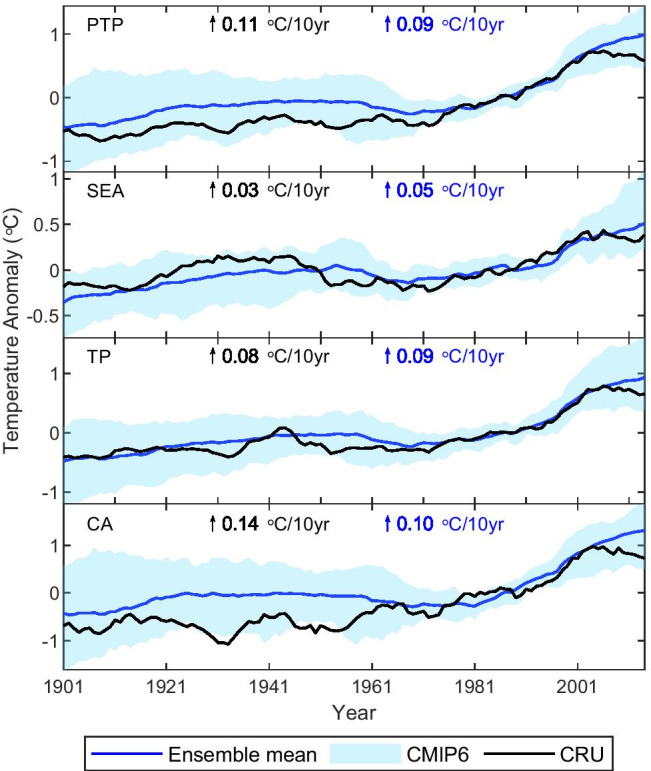
Time series of 10-year moving average annual surface mean temperature from the CMIP6 models and CRU observational dataset over the PTP region and its three subregions during 1901–2014 relative to the period 1970–1999 (blue line and shading: CMIP6; black line: CRU). The trends are calculated for the observations and the CMIP6 ensemble mean during 1901–2014. The shading indicates the ensemble spread (range between the 5th and 95th quantiles)

Figure 4 shows 10-year moving average values for annual mean temperature for the ensemble of the 16 models and for the observations. The analysis shows that the observed annual mean temperature lies within the 5th–95th percentile range of CMIP6 multi-model ensembles, implying that there is consistency between the observed record and the CMIP6 models. Additionally, the CMIP6 historical simulations can reproduce the observed annual temperature warming trends in the PTP region and its three subregions, although with different magnitudes for bias.

### Evaluations of historical temperature extremes

**Fig. 5:**
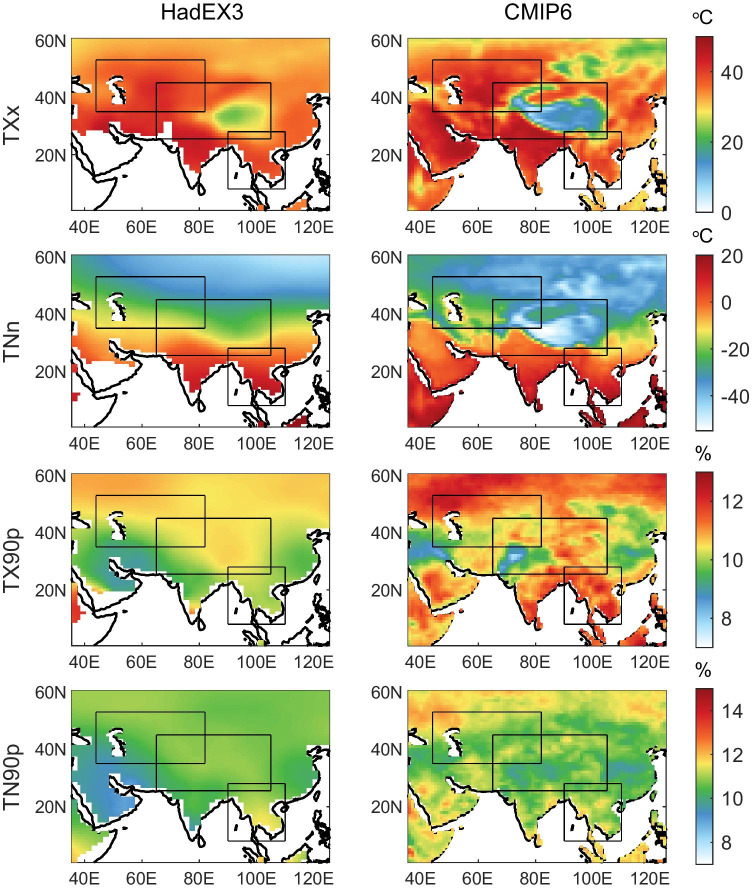
Spatial distributions of annual mean max TX (TXx), min TN (TNn), warm days (TX90p), and warm nights (TN90p) for HadEX3 and the CMIP6 ensemble mean over the PTP region for the 1970–1999 average

The spatial distributions of the four annual mean temperature extreme indices (TXx, TNn, TX90p, and TN90p) from HadEX3 and the CMIP6 multi-model mean during 1970–1999 over the PTP region are shown in Fig. 5. In general, the CMIP6 ensemble mean could capture the key features of the spatial patterns of temperature extremes effectively. We also investigate the spatial variability of climatological annual mean temperature extreme indices for 12 individual models, and the results are shown in Figures S1 to S4. For TXx, the multi-model mean and each model display consistent positive biases in most regions but show a negative bias in the TP. MIROC6 shows a larger warm bias than other models, but a smaller bias in the TP region. Among the four extreme indices, the CMIP6 models perform best in simulating TNn, with considerable consistency across models. However, most models underestimate the observed TNn of the TP. The TX90p for the CMIP6 models and the multi-model mean displays substantially positive biases of about 2% over most parts of the PTP region, especially in the northern parts of the PTP region and South Asia. Several models (especially EC-Earth3-Veg, EC-Earth3, CanESM5, GFDL-ESM4, and IPSL‐CM6A‐LR, and MRI-ESM2-0) simulate high TN90p over parts of the PTP region. These biases are also found in the CMIP6 multi-model mean, but with smaller magnitude. In general, the four extreme indices simulated by the CMIP6 models are basically biased in the TP region, which may be due to the lack of observation stations and complex terrain in this region, with the coarse resolutions of the climate models leading to difficulties in the model simulation (You et al. 2018; Zhu and Yang 2020).

**Fig. 6:**
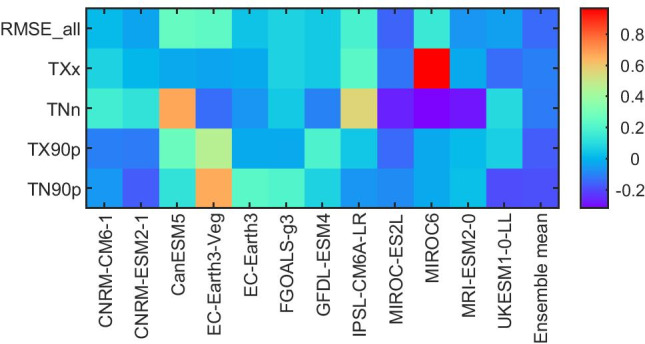
The portrait diagram of relative RMSEs for the 1970–1999 climatologies of temperature extreme indices simulated by the CMIP6 models with respect to the HadEX3 dataset. (Note that the models were masked by HadEX3 due to the insufficient spatial coverage of HadEX3.) The RMSEs are spatially averaged over the PTP region and the top row indicates the mean relative RMSE across all indices for a particular model

A portrait diagram is a powerful tool for evaluating individual models against a reference dataset. Figure 6 presents a portrait diagram for the chosen temperature extreme indices, displaying the relative magnitude of spatially averaged RMSE and the average RMSE for all indices (RMSE_all) in the top row. The cold colors indicate when a model’s performance is better than others’, on average, and the warm colors indicate when its performance is worse. In general, most models shown exhibit reasonable skill in representing the temperature-based indices at the annual scale, while the performance of several models deviates greatly from that of the median model with respect to HadEX3 (e.g., MIROC6 for TXx, CanESM5 and IPSL‐CM6A‐LR for TNn, EC-Earth3-Veg for TN90p and TN90p). Models that perform relatively well for these four indices include MIROC-ES2L, MRI-ESM2-0, UKESM1-0-LL, CNRM-CM6-1, and CNRM-ESM2-1. Based on the RMSE_all (top raw), MIROC-ES2L shows the best performance, with negative relative RMSEs for all four indices, followed by MRI-ESM2-0, UKESM1-0-LL, and CNRM-ESM2-1, which exhibit relatively small RMSE′ of around 0. The performance of the multi-model mean is superior to that of most individual models due to substantial reduction of the systematic errors in individual models (Kim et al. 2020; Sillmann et al. 2013).

**Fig. 7:**
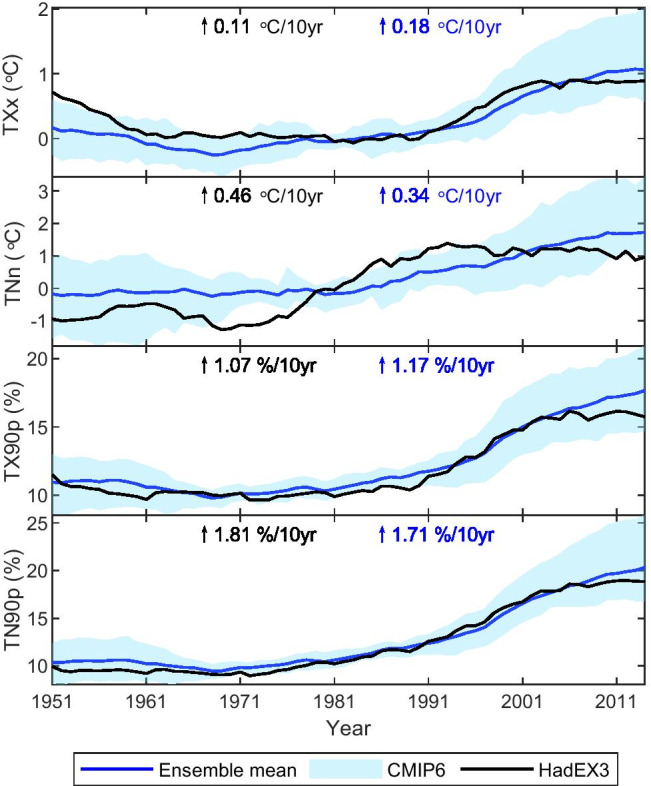
Time series of the 10-year moving average annual mean max TX (TXx), min TN (TNn), warm days (TX90p), and warm nights (TN90p) from the CMIP6 models and the HadEX3 observational dataset over the PTP region during 1951–2014. For TXx and TNn, the time series shows anomalies with respect to the reference period 1970–1999; for TX90p and TN90p, the time series are displayed as absolute exceedance rate. The trends are calculated for the observations and the CMIP6 ensemble mean during 1951–2014. The shading indicates the ensemble spread (range between the 5th and 95th quantiles)

The temporal evolution of the regional averaged indices over the PTP region in the models and HadEX3 is shown in Fig. 7 for the time period 1951–2014, and the time series of the three subregions are shown in Figures S5–S7. Note that we use HadEX3 to mask the CMIP6 models to avoid effects related to changes in the spatial coverage of the HadEX3 dataset over time. We found that the HadEX3 temperature indices generally lie within the CMIP6 model spread, with similar variability in many cases. Some exceptions are TNn for whole PTP region and its subregions (Figs. 7, S5–S7), and TXx before 1960s for the TP region (Figure S6). For all four indices, there are similar warming trends in the models and HadEX3 for the entire PTP region and the three subregions (except TXx in TP, which may be due to the lack of observed stations in TP). For the extreme indices based on the TX (TXx and TX90p), the trend simulation of the CMIP6 multi-model average generally overestimates the warming trend of the observations, while for the extremes indices based on the TN (TNn and TN90p), the CMIP6 multi-model average shows a lower increase compared to the HadEX3 dataset.

## Projected temperatures in the twenty-first century

### Projected changes in mean temperatures

**Fig. 8:**
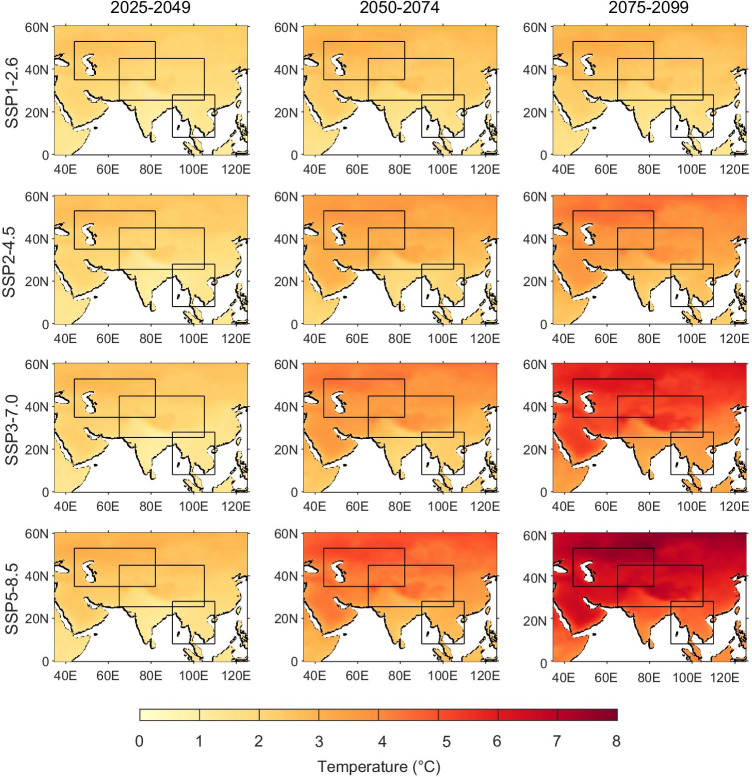
Spatial distribution of changes in annual mean temperatures over the PTP region in the near-term (2025–2049), mid-term (2050–2074), and long-term (2075–2099) periods of the twenty-first century, relative to 1970–1999, under the SSP1-2.6, SSP2-4.5, SSP3-7.0, and SSP5-8.5 scenarios

In this section, we present the projected changes of temperature over the PTP region in the twenty-first century under the scenarios of SSP1-2.6, SSP2-4.5, SSP3-7.0, and SSP5-8.5. We found that the performance of the model is inconsistent for different temperature variables. However, the multi-model means generally exhibit better performance than most individual models, so we use the multi-model mean for projections of the future. Figure 8 shows the spatial distributions of climatological changes in mean temperature in terms of multi-model ensemble means for the near-term (2025–2049), mid-term (2050–2074), and long-term (2075–2099) periods of the twenty-first century, compared to the baseline period (1970–1999). Projected changes in annual mean temperature suggest increasingly widespread temperature increases under the four future scenarios across the whole PTP region. The largest increases of temperature are predicted for the northern parts of the PTP region—Central Asia and the Tibetan Plateau. In the near term, the different emission scenarios do not lead to dramatically different temperature responses and the multi-model mean projects a less than 3 °C increase relative to the reference period under the four scenarios. The mid-term period can be regarded as a transition period during which the different temperature responses for the SSP2-4.5, SSP3-7.0, and SSP5-8.5 scenarios become increasingly noticeable, with maximum values of 3.87 °C, 4.55 °C, and 5.28 °C, respectively. However, the temperature changes under the low-forcing sustainability pathway (SSP1-2.6 scenario) are relatively small throughout the twenty-first century, with increases generally remaining within 3 °C. By the end of the twenty-first century (2075–2099), changes under the SSP5-8.5 are much larger than other scenarios. The increase in the annual mean temperature under the high‐forcing pathway (SSP5-8.5) will exceed 6 °C over most of the PTP region and will exceed 7 °C over the Tibetan Plateau and Central Asia.

**Fig. 9:**
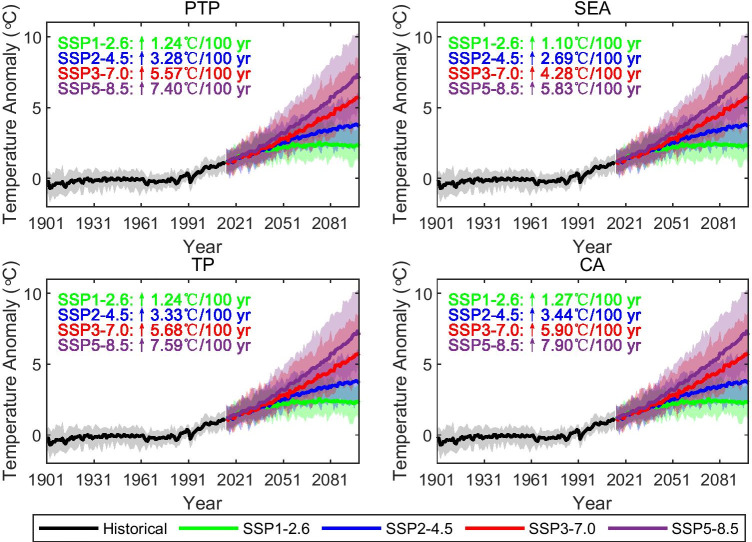
Time series of changes in annual mean temperature over the PTP region and its three subregions during 1901–2099 relative to the period 1970–1999. The black, green, blue, red, and purple curves represent the results of the CMIP6 ensemble mean for the historical period and for the SSP1-2.6, SSP2-4.5, SSP3-7.0, and SSP5-8.5 scenarios, respectively. The shaded areas are the spreads from the 5th to the 95th percentiles of the annual mean temperatures

The time series of annual mean temperature in the historical (1901–2014) and projected periods (2015–2099) in the PTP region, SEA, TP, and CA are illustrated in Fig. 9. The simulations of the multi-model mean show that the annual mean temperature of the PTP region and all three subregions will increase remarkably over the twenty-first century under all scenarios. On average, the temperature over the whole PTP region will rise by 1.24 °C/100 year, 3.28 °C/100 year, 5.57 °C/100 year, and 7.40 °C/100 year for the SSP1-2.6, SSP 2–4.5, SSP3-7.0, and SSP5-8.5 scenarios, respectively. The projected warming trends under SSP5-8.5 are considerably higher than those under SSP1-2.6 in all the considered regions. The highest warming trends occur in the CA region under all four scenarios, following by the TP region, while the lowest warming trends appear in SEA. The strong warming trends in the entire PTP region, the CA region, and the TP region are all higher than the global land average (about 7.20 °C/100 year under SSP5-8.5) (Fan et al. 2020a), which will cause greater threats in the vulnerable ecological systems of these regions. Additionally, we found that the warming trend slows down or even declines after 2050 under SSP1-2.6 for the PTP region and its three subregions. For the model uncertainty of the mean temperature projections, the results suggested that the uncertainty increases with time in the twenty-first century, and the uncertainty under high-forcing pathways is larger than it is under medium- and low-forcing pathways.

### Projected changes in temperature extremes

**Fig. 10:**
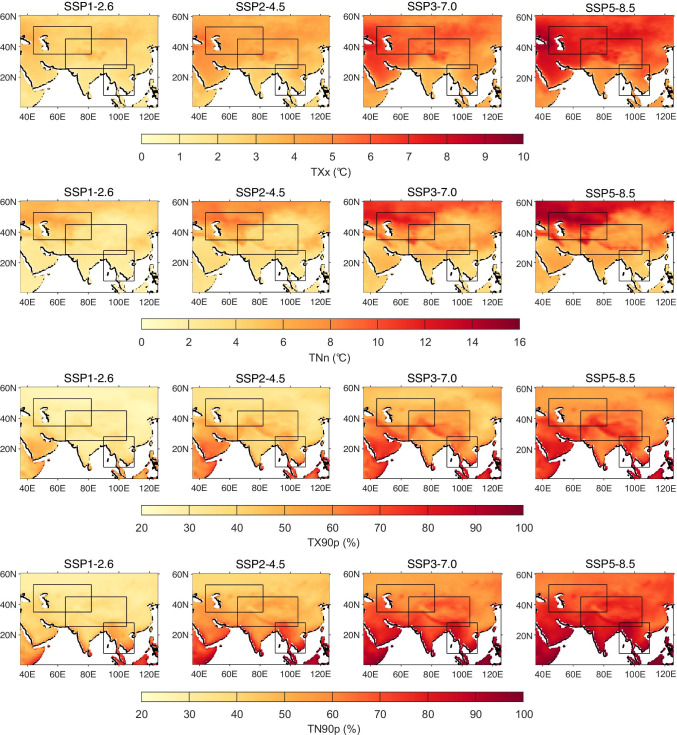
Spatial distributions of projected changes in annual mean max TX (TXx), min TN (TNn), warm days (TX90p), and warm nights (TN90p) over the period 2075–2099 relative to the reference period 1970–1999 under the SSP1-2.6, SSP2-4.5, SSP3-7.0, and SSP5-8.5 scenarios. Note that TX90p and TN90p are displayed as absolute exceedance rate

Figure 10 presents the spatial distributions of projected changes in temperature extreme indices over the PTP region during the long-term future period at the end of the twenty-first century (2075–2099). The spatial distributions of changes in the near-term (2025–2049) and mid-term (2050–2074) periods are shown in Figures S8 to S11. Projected changes in the TX90p and TN90p are shown in absolute values, and not as differences relative to the reference period as for the TXx and TNn. This is because the percentile indices represent exceedance rates (in %) relative to the 1961–1990 base period, which has been used as the baseline for future changes. All four extreme temperature indices are projected to show prominent increases in the PTP region, with stronger warming under the SSP5-8.5 scenario (Figures S8–S11). The most intense warming for TXx and TNn is mainly projected in the CA region, with magnitudes of around 8 °C and 14 °C, respectively. A pronounced increase in warm days (TX90p) is projected over most of the TP region under all SSP scenarios (around 80% for SSP5-8.5), and the index value in the SEA region will also increase greatly by the end of the twenty-first century. For warm nights (TN90p), the projected strongest increases occur in the SEA region, where the exceedance rate in some areas reaches 99.6%. The robustness of the projected increases of these four indices over the entire PTP region suggests a potential risk of intensified temperature extremes to natural and social systems under the accelerated emission scenarios. Nevertheless, consistent with the changes in mean temperatures described above, the extreme temperature indices also show little difference with time under the SSP1-2.6 scenario, which indicates the effectiveness of anticipated climate mitigation and adaptation strategies associated with this scenario.

**Fig. 11:**
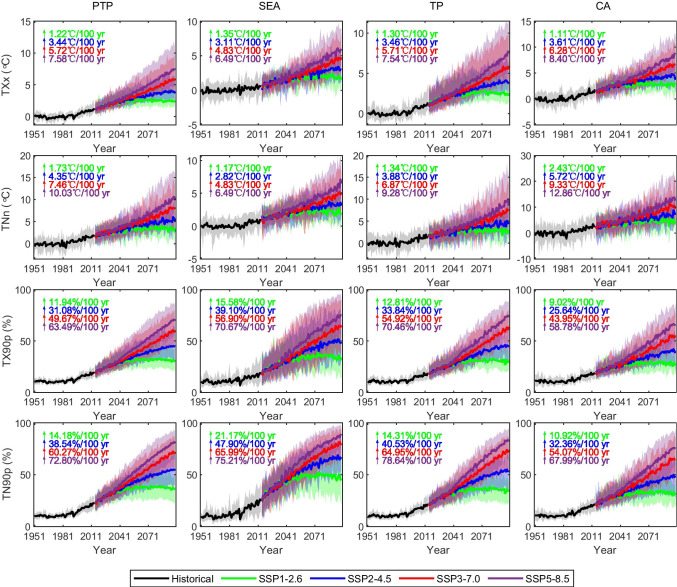
Time series of changes in annual mean max TX (TXx), min TN (TNn), warm days (TX90p), and warm nights (TN90p) over the PTP region and its three subregions during 1901–2099 relative to the period 1970–1999. (Note that the time series for TX90p and TN90p are displayed as an absolute exceedance rate.) The black, green, blue, red, and purple curves represent the results for the CMIP6 ensemble mean for the historical period and for the SSP1-2.6, SSP2-4.5, SSP3-7.0, and SSP5-8.5 scenarios, respectively. The shaded areas are the spreads from the 5th to the 95th percentiles of the annual mean temperature extreme indices

To identify the inter-annual trend under different scenarios, the time series of regional average annual temperature extremes indices over the three subregions and the entire PTP region during 1951–2099 are shown in Fig. 11. In general, the CMIP6 models exhibit increasing trends in annual TXx, TNn, TX90p, and TN90p over the PTP region and its three subregions in the twenty-first century. The increase in TXx is lower than that in TNn for all four CMIP6 SSPs (except SEA region). For the PTP region, the multi-model mean increases in TXx and TNn, respectively, that are projected by the end of the twenty-first century are 1.22 °C/100 year and 1.73 °C/100 year in SSP1-2.6, 3.44 °C/100 year and 4.35 °C/100 year in SSP2-4.5, 5.72 °C/100 year and 7.46 °C/100 year in SSP3-7.0, and 7.58 °C/100 year and 10.03 °C/100 year in SSP5-8.5. By the end of the twenty-first century, the greatest warming trends under the four scenarios for TXx and TNn are projected in the CA region (8.40 °C/100 year and 12.86 °C/100 year in SSP5-8.5, respectively). The increasing trends of TN90p are greater than those of warm days (TX90p). Toward the end of the twenty-first century, the warming trends for TX90p and TN90p over the entire PTP region are 11.94%/100 year and 14.18%/100 year for SSP1-2.6, 31.08%/100 year and 38.54%/100 year for SSP2-4.5, 49.67%/100 year and 60.27%/100 year for SSP3-7.0, and 63.49%/100 year and 72.80%/100 year for SSP5-8.5. The strongest warming for TX90p occurs in the SEA region (70.67%/100 year) followed by warming in the TP region of about 70.46%/100 year for SSP5-8.5. The highest increase for TN90p (about 78.64%/100 year for SSP5-8.5 by year 2099) occurs in the TP region, followed by the SEA region (75.21%/100 year). In conclusion, the warming trends of the nighttime extremes (TNn and TN90p) are larger than those of the daytime extremes (TXx and TX90p), which is consistent with the results for CMIP5 in previous studies (Yin et al. 2019; You et al. 2018), probably because the water vapor and radiative feedbacks are enhanced at lower air temperatures (Ohmura 2012; You et al. 2018).

## Summary and conclusions

In this work, a comprehensive evaluation of CMIP6 ensembles over the Pan-Third Pole region was performed to assess their performance in simulating spatial patterns and temporal variability of mean temperatures and four indices of temperature extremes, which constitute the most important parameters for modeling climate-related changes to the terrestrial water cycle. From the results of the spatial pattern analysis of the mean and extreme temperatures, we found that most CMIP6 simulations are in fairly good agreement with CRU or HadEX3 observations in many areas, but model replication of observed temperature patterns over the Tibetan Plateau is problematic. The multi-model ensemble mean is found to be superior to most CMIP6 model simulations, overall.

Then, temperature projections for the twenty-first century were estimated for four integrated scenarios of socioeconomic development and greenhouse gas emissions, SSP1-2.6, SSP2-4.5, SSP3-7.0, and SSP5-8.5. The multi-model ensemble of CMIP6 models reveals a continuous increase in the annual mean temperature and four extremes indices over the PTP region during the twenty-first century under all four SSP scenarios. The northern parts of PTP (Central Asia and the Tibetan Plateau) are projected to experience the largest increases in future mean temperature (exceeding 7 °C) by the end of the twenty-first century under SSP5-8.5 relative to the reference periods (1970–1999). For TXx and TNn, the most intense warming will occur in the CA region, with magnitudes of around 8 °C and 14 °C, respectively. The pronounced increase of warm days (TX90p) is projected to be around 80% over the TP region and under the SSP5-8.5 scenario, and the greatest number of projected warm nights (TN90p) occur in the SEA region, where the exceedance rate in some areas reaches 99.6%.

By the end of the twenty-first century, the annual mean temperature averaged over the PTP region is projected to increase for the SSP1-2.6, SSP 2–4.5, SSP3-7.0, and SSP5-8.5 scenarios by 1.24 °C/100 year, 3.28 °C/100 year, 5.57 °C/100 year, and 7.40 °C/100 year, respectively. For temperature extremes, we found that the increasing trends in indices based on TN are greater than the increases in indices based on TX for all four CMIP6 SSPs. The CA region is projected to exhibit the greatest warming trends for TXx and TNn across the four scenarios (8.40 °C/100 year and 12.86 °C/100 year for SSP5-8.5, respectively). The strongest warming during the twenty-first century for TX90p and TN90p occurs during SSP5-8.5 in the SEA region (70.67%/100 year) and the TP region (78.64%/100 year), respectively. Finally, we note that the projected changes in mean and extreme temperatures are stronger under the highest emissions scenario (SSP5-8.5). However, the temperatures start to decrease under the SSP1-2.6 scenarios around 2080, which largely reflect the design of the downward trajectories in terms of socioeconomic development and radiative forcing in SSP1-2.6 scenario (Gidden et al. 2019). This indicates that the future heat risk in the Pan-Third Pole region would be mitigated by reducing greenhouse gas emissions. Although risks will be reduced substantially under the low emissions scenario (SSP1-2.6) with the lowest temperature projections compared to the highest temperature projections (SSP5-8.5–high emissions), the rising temperatures still have non-negligible impacts on the ecological environment of the Pan-Third Pole region, such as glacial melting, grassland degradation, soil erosion, and natural disaster. Practical adaptation measures are essential to reduce vulnerability to the negative effects of climate change. To effectively cope with climate change impacts, a regional integrated water resource management approach is needed. Important factors such as soil health, erosion, and land use management should be taken into account in order to improve agricultural productivity and hydropower production while protecting natural resources (Kong et al. 2022). The alpine grassland water-saving irrigation was recommended as key measure and supplemented with reasonable grazing management, alpine grassland fencing, and artificial grass planting measures. In order to better response with natural disasters, the government needs to pay more attention to the hotspot areas within the plateau most sensitive to the climate change, strengthen scientific research in these areas, and enhance monitoring, regulation, and warning systems. Greater international cooperation is also needed to effectively adapt to climate change and mitigate its effects.

## Supplementary Information

Below is the link to the electronic supplementary material.

Supplementary file1 (DOCX 3808 kb)

## Data Availability

The datasets used and/or analyzed during the current study are available from the corresponding author on reasonable request.
