# Supplementary material for: Evaluation of historical CMIP6 model simulations and future projections of temperature over the Pan-Third Pole region
Source: Environ Sci Pollut Res Int. 2021 Dec 1;29(18):26214–29. doi: 10.1007/s11356-021-17474-7 (PMC8989916; doi:10.1007/s11356-021-17474-7)
Supplement: Supplementary file 1 — Supplementary file1 (DOCX 3808 kb) [file 11356_2021_17474_MOESM1_ESM.docx]

*Supporting Information for*

**Evaluation of historical CMIP6 model simulations and future projections of temperature over the Pan-Third Pole region**

Contents of this file

Figures S1 to S11


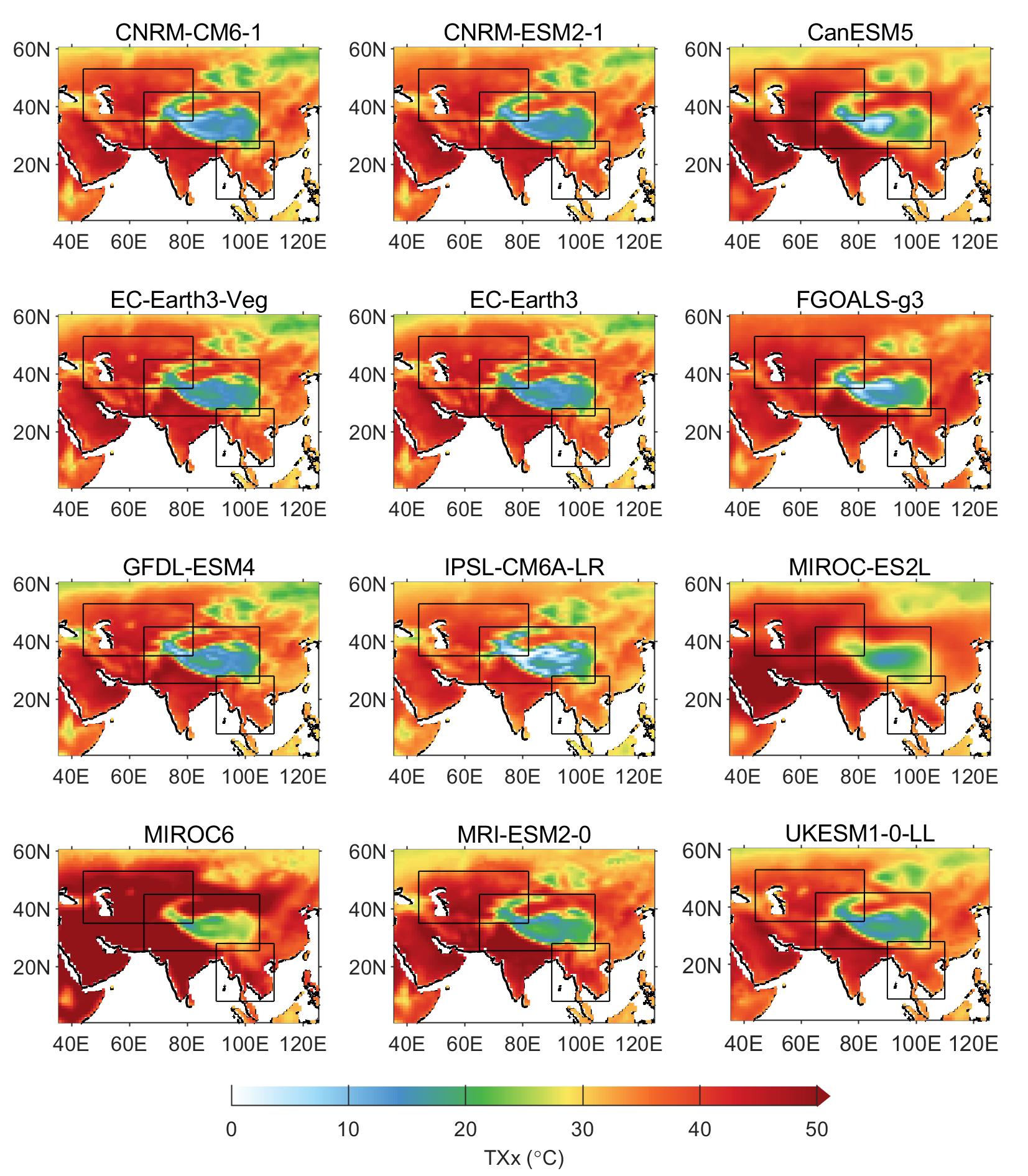


Figure S1 Spatial distributions of annual mean max TX (TXx) for the CMIP6 models over the PTP for the 1970–1999 average.


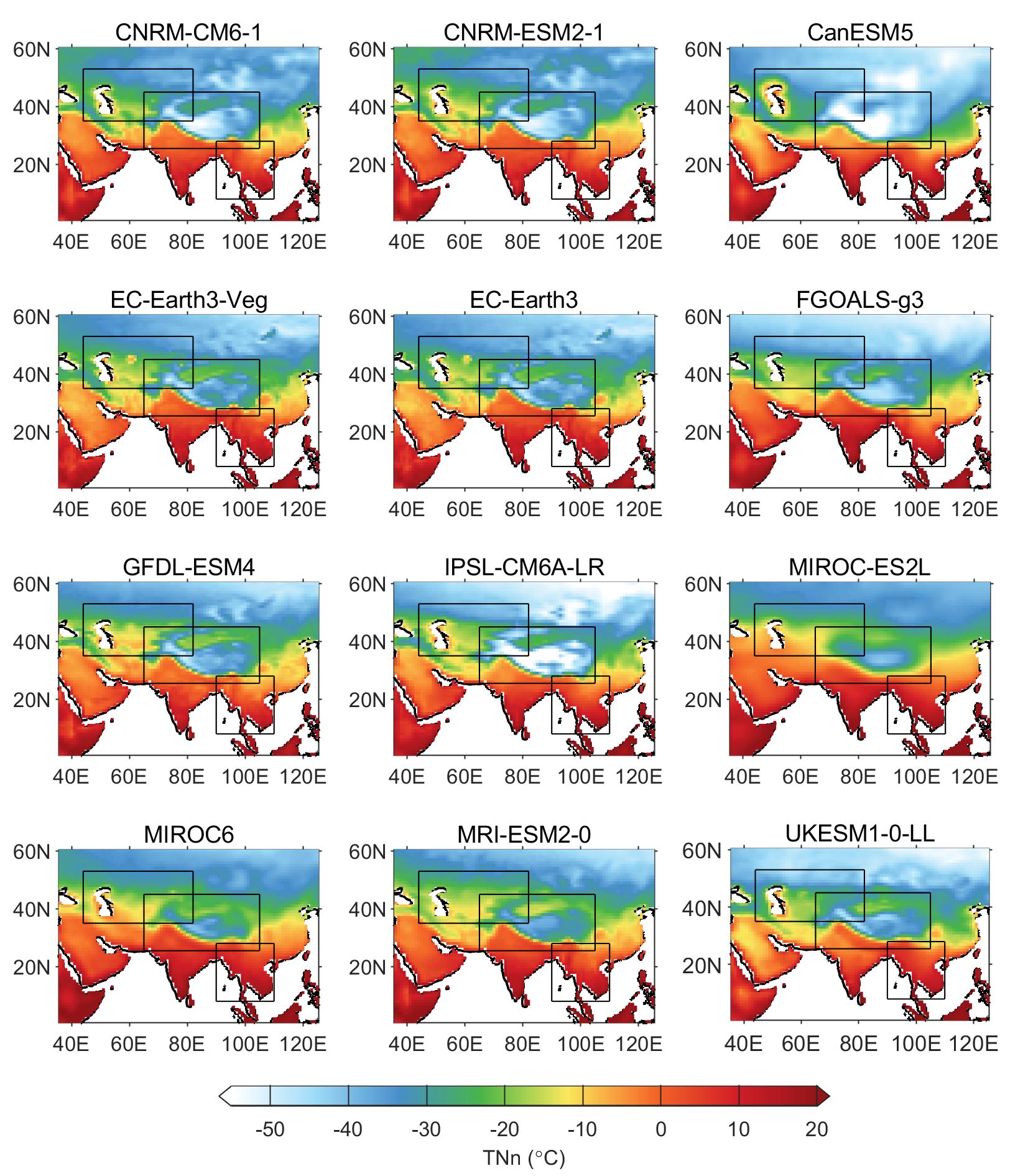


Figure S2 Spatial distributions of annual mean min TN (TNn) for the CMIP6 models over the PTP for the 1970–1999 average.


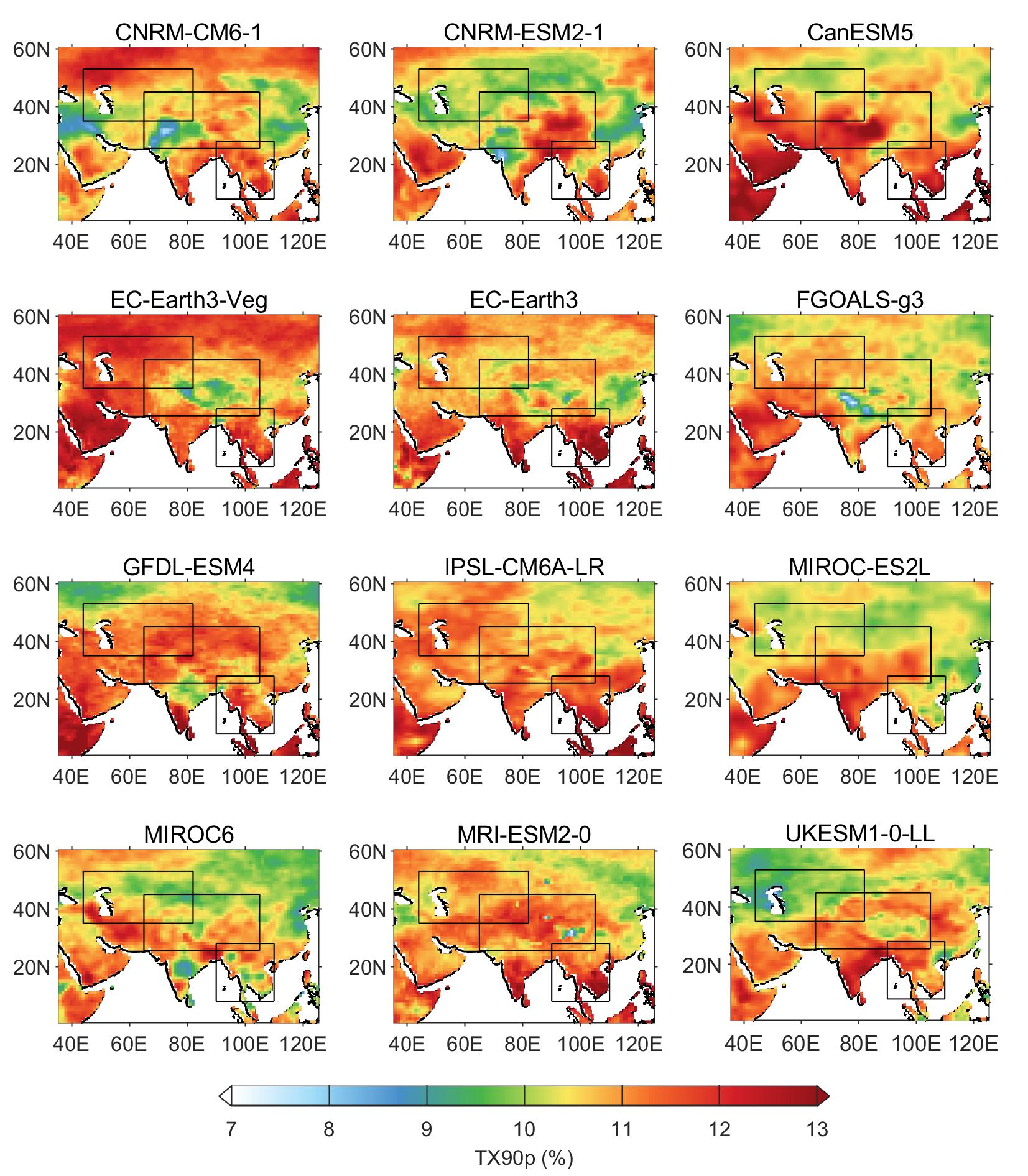


Figure S3 Spatial distributions of annual mean warm days (TX90p) for the CMIP6 models over the PTP for the 1970–1999 average.
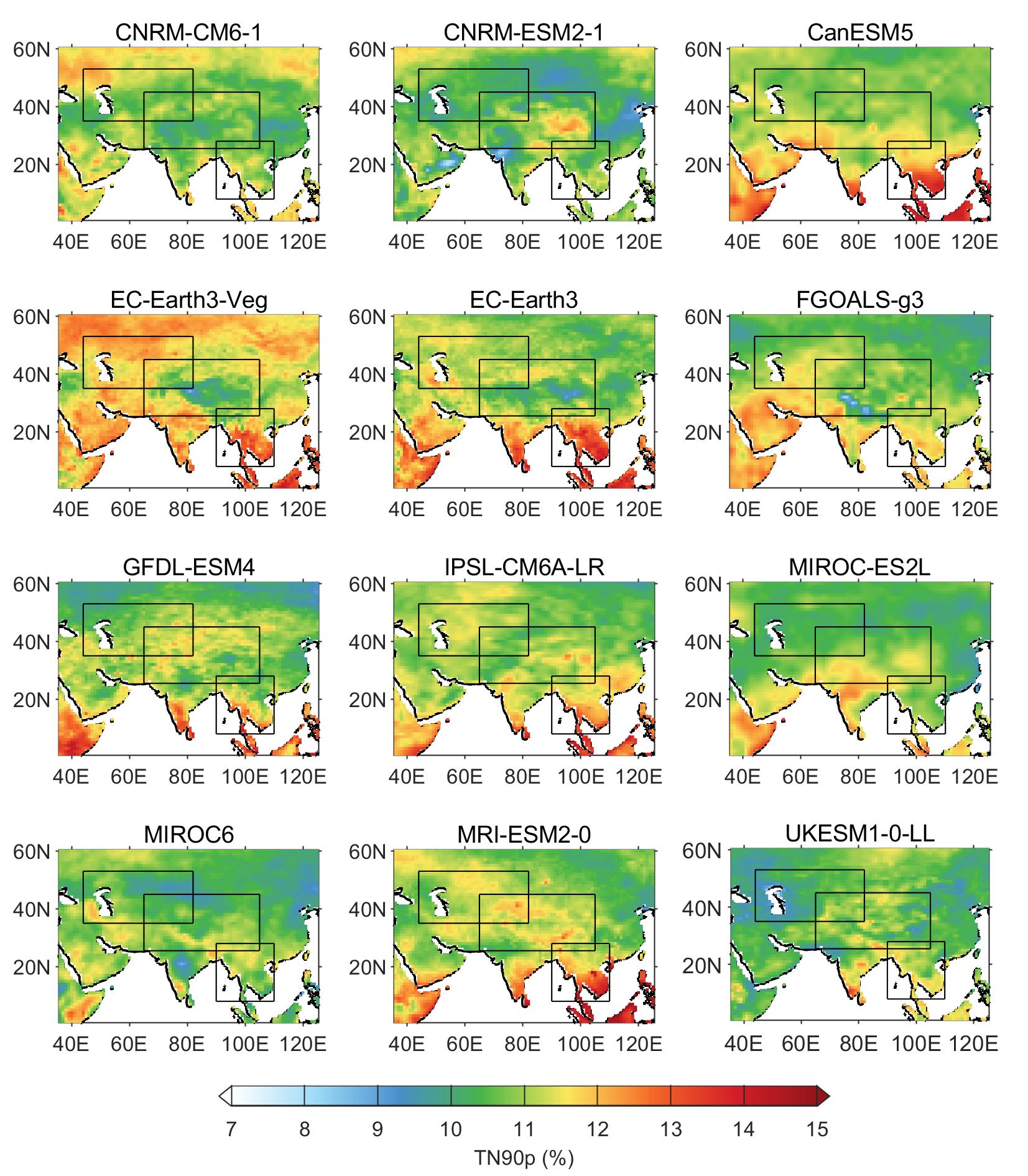


Figure S4 Spatial distributions of annual mean warm nights (TN90p) for the CMIP6 models over the PTP for the 1970–1999 average.


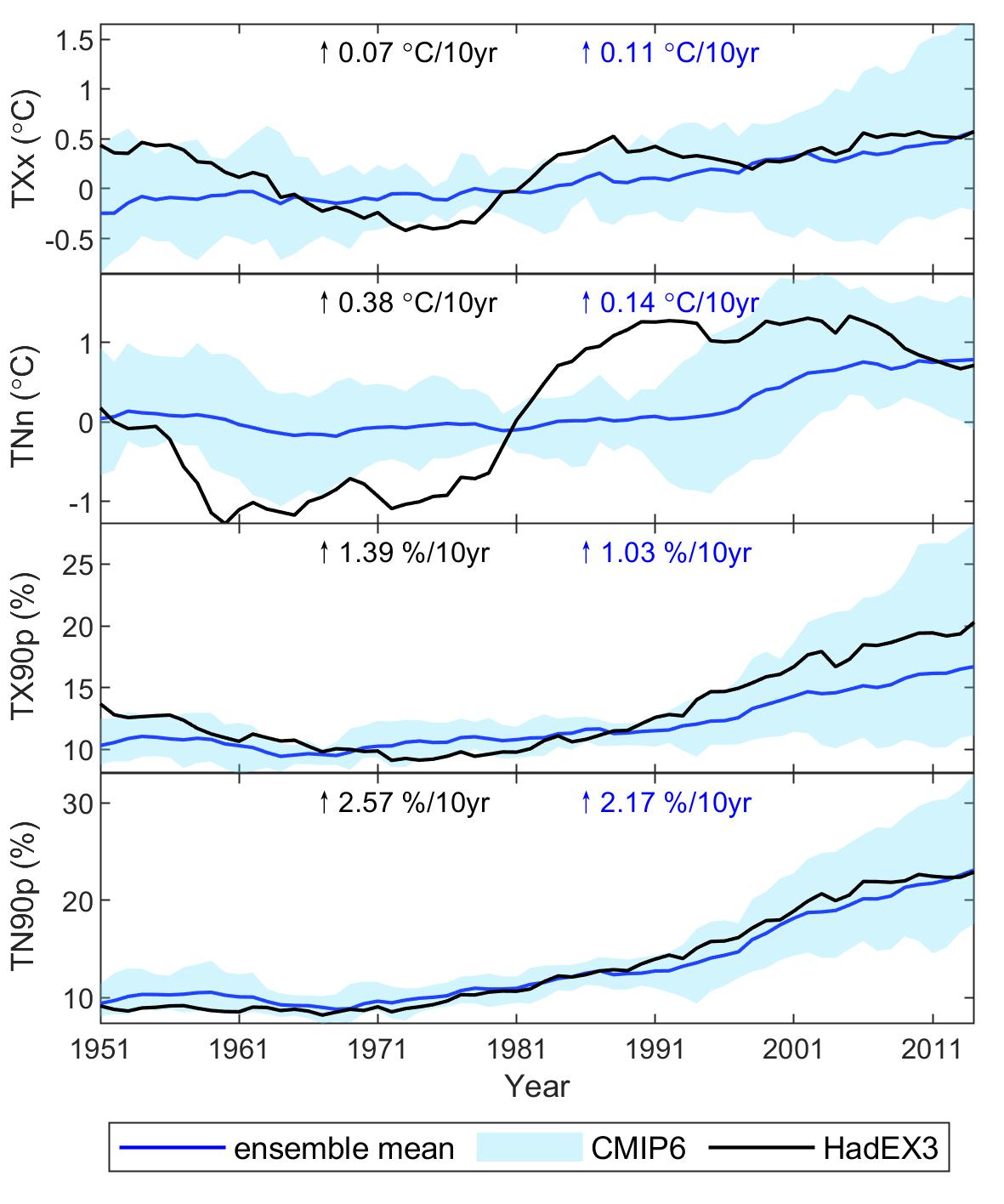


Figure S5 Time series of 10-year moving average annual mean max TX (TXx), min TN (TNn), warm days (TX90p), and warm nights (TN90p) from the CMIP6 models and HadEX3 observational dataset over the SEA region during 1951–2014 (for TXx and TNn, the time series shows anomalies with respect to the reference period 1970 to 1999; for TX90p and TN90p, the time series are displayed as absolute exceedance rates). The trends are calculated for the observations and the CMIP6 ensemble mean during 1951–2014. The shading indicates the ensemble spread (range between the 5th and 95th quantiles).


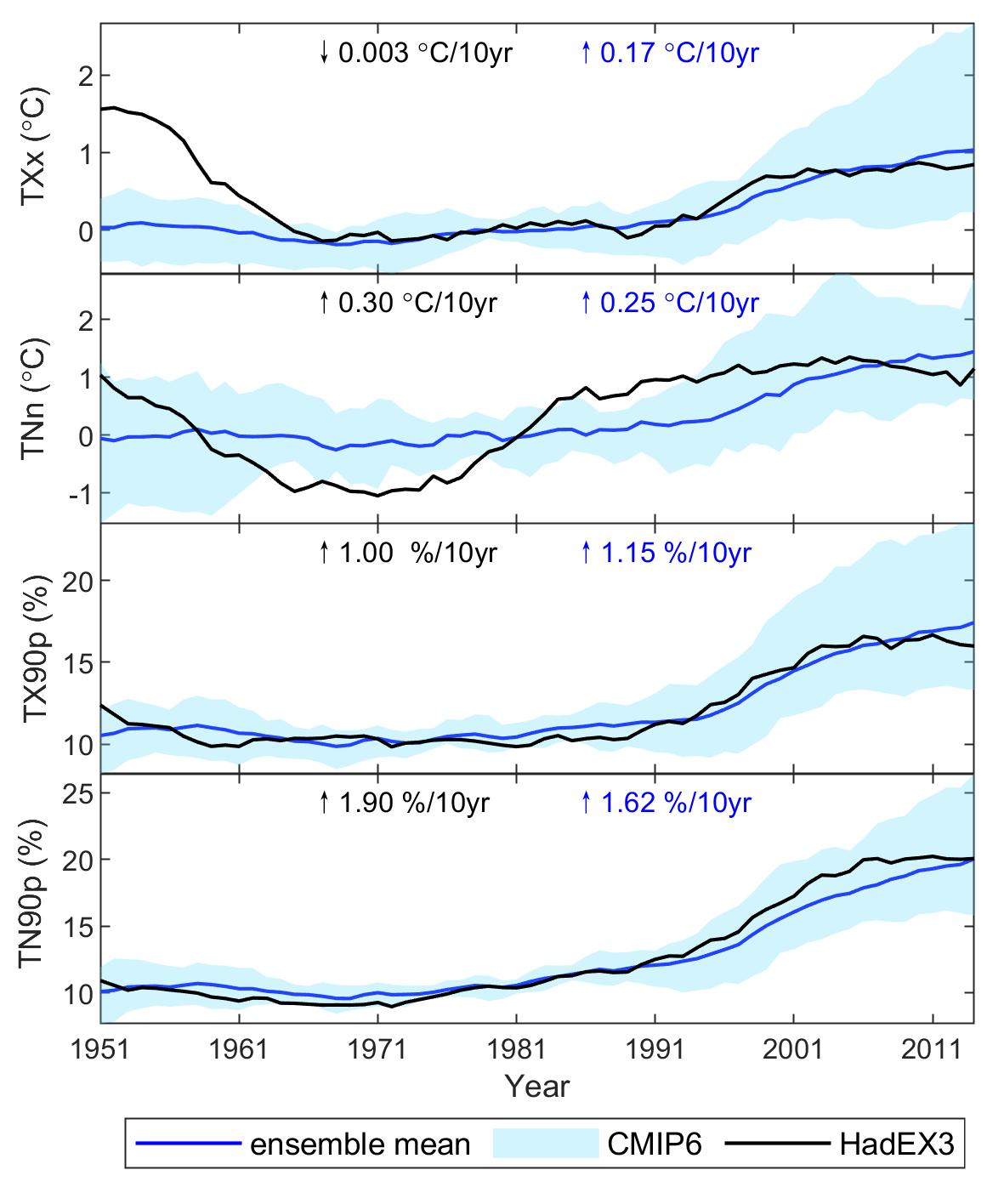


Figure S6 Same as Figure S5, but for the TP region.


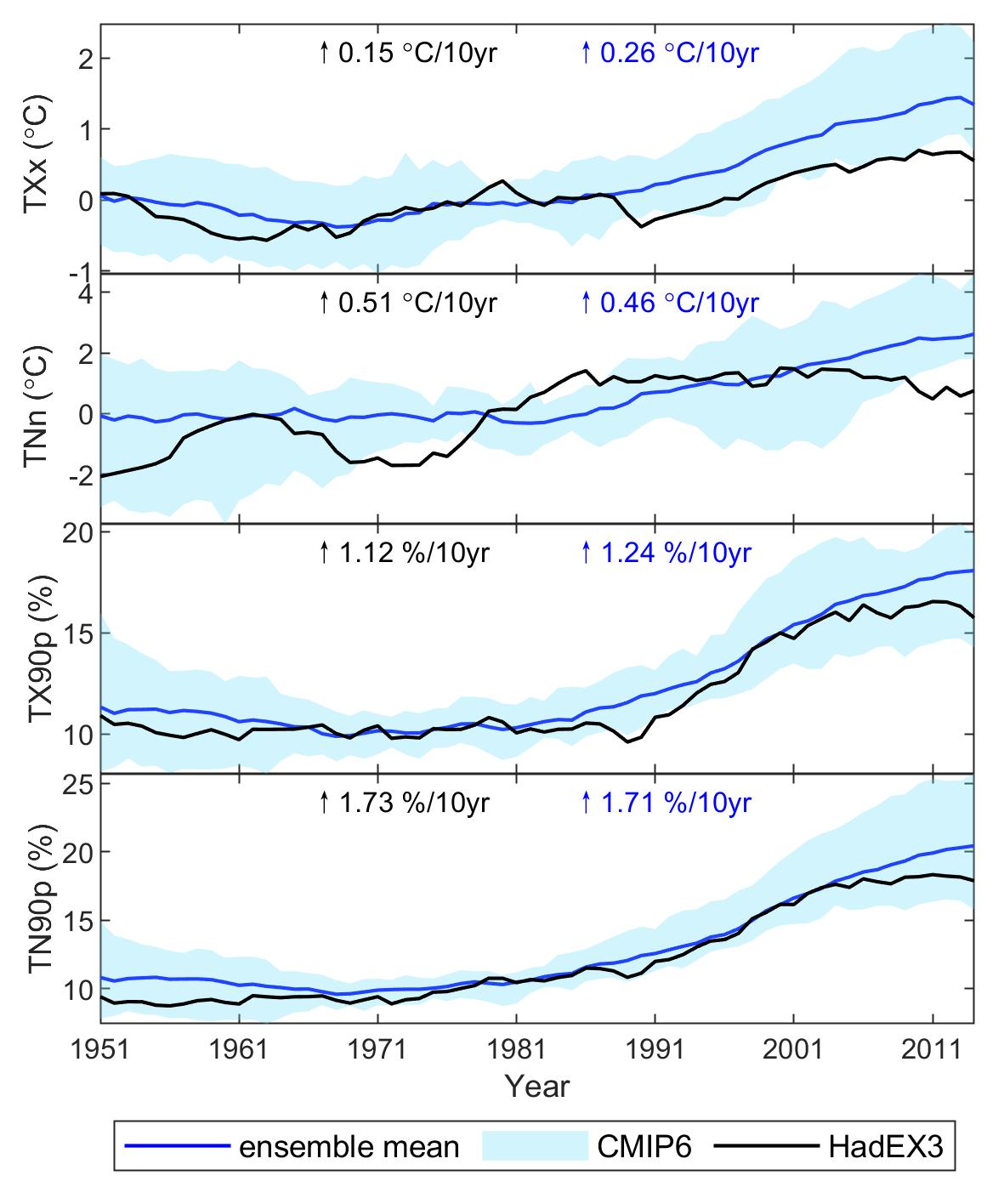


Figure S7 Same as Figure S5, but for the CA region.


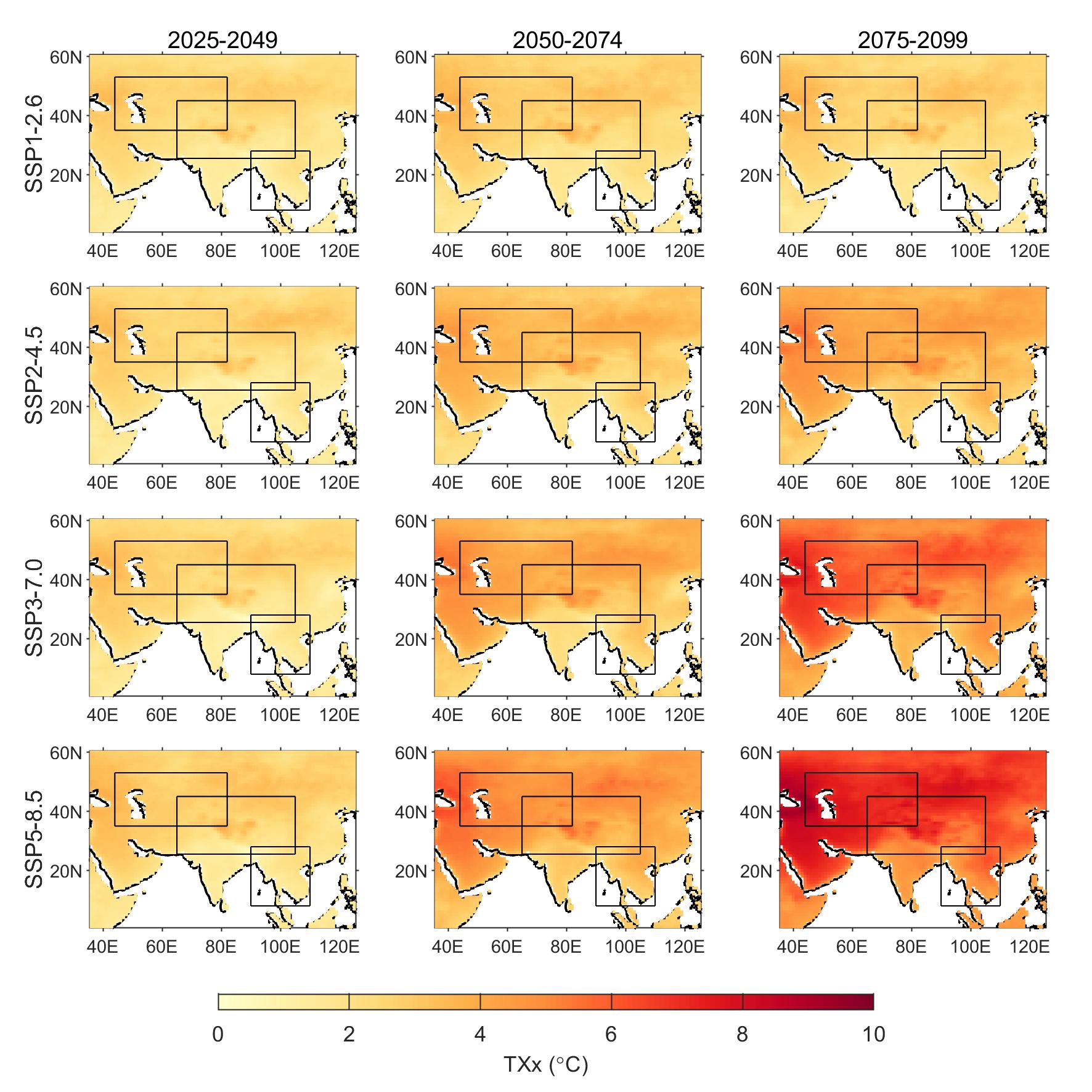


Figure S8 Spatial distributions of changes in annual mean max TX (TXx) over the PTP regions in near-term (2025–2049), mid-term (2050–2074), and long-term (2075–2099) periods of the 21st century, relative to 1970–1999, under the SSP1-2.6, SSP2-4.5, SSP3-7.0, and SSP5-8.5 scenarios.


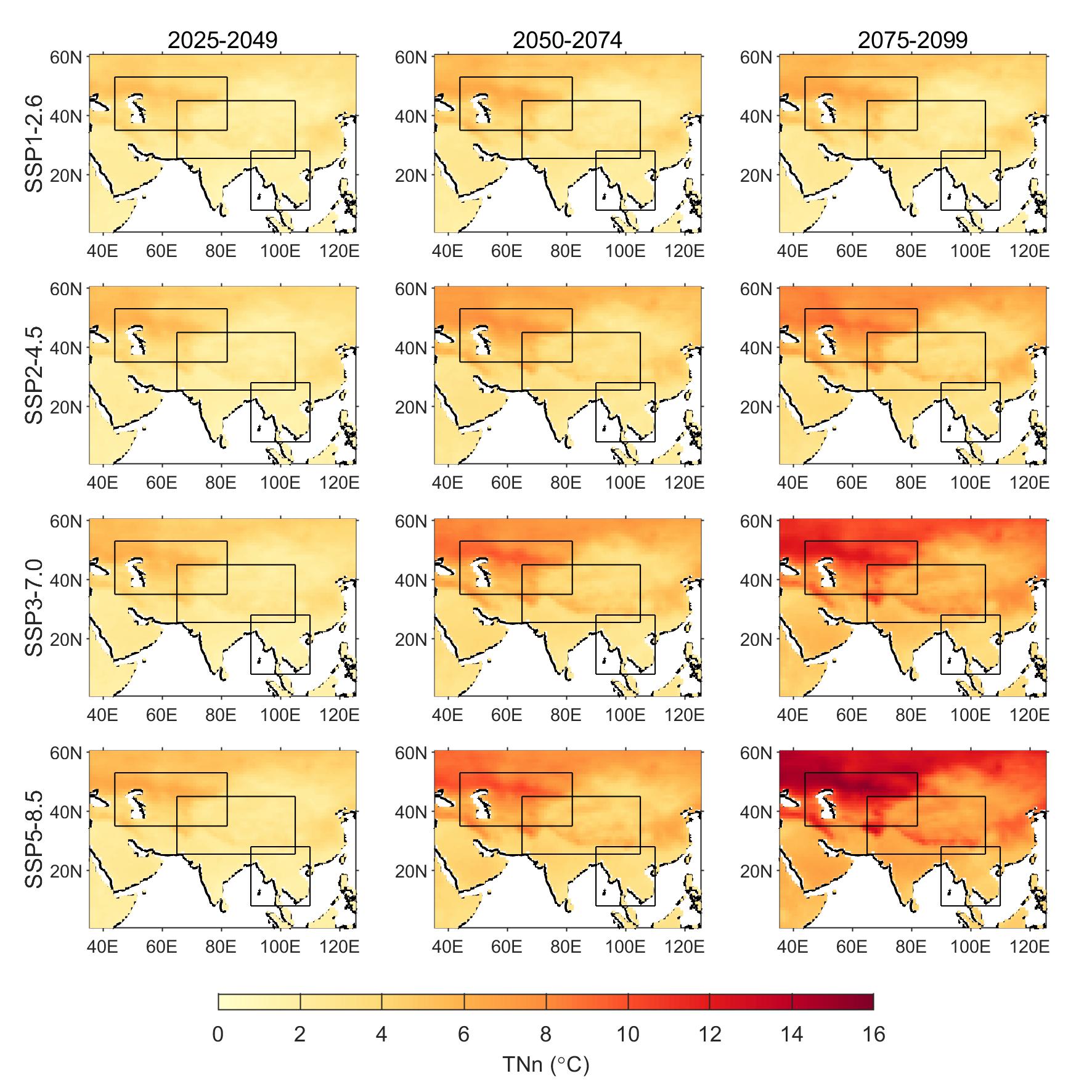


Figure S9 Same as Figure S8, but for min TN (TNn).


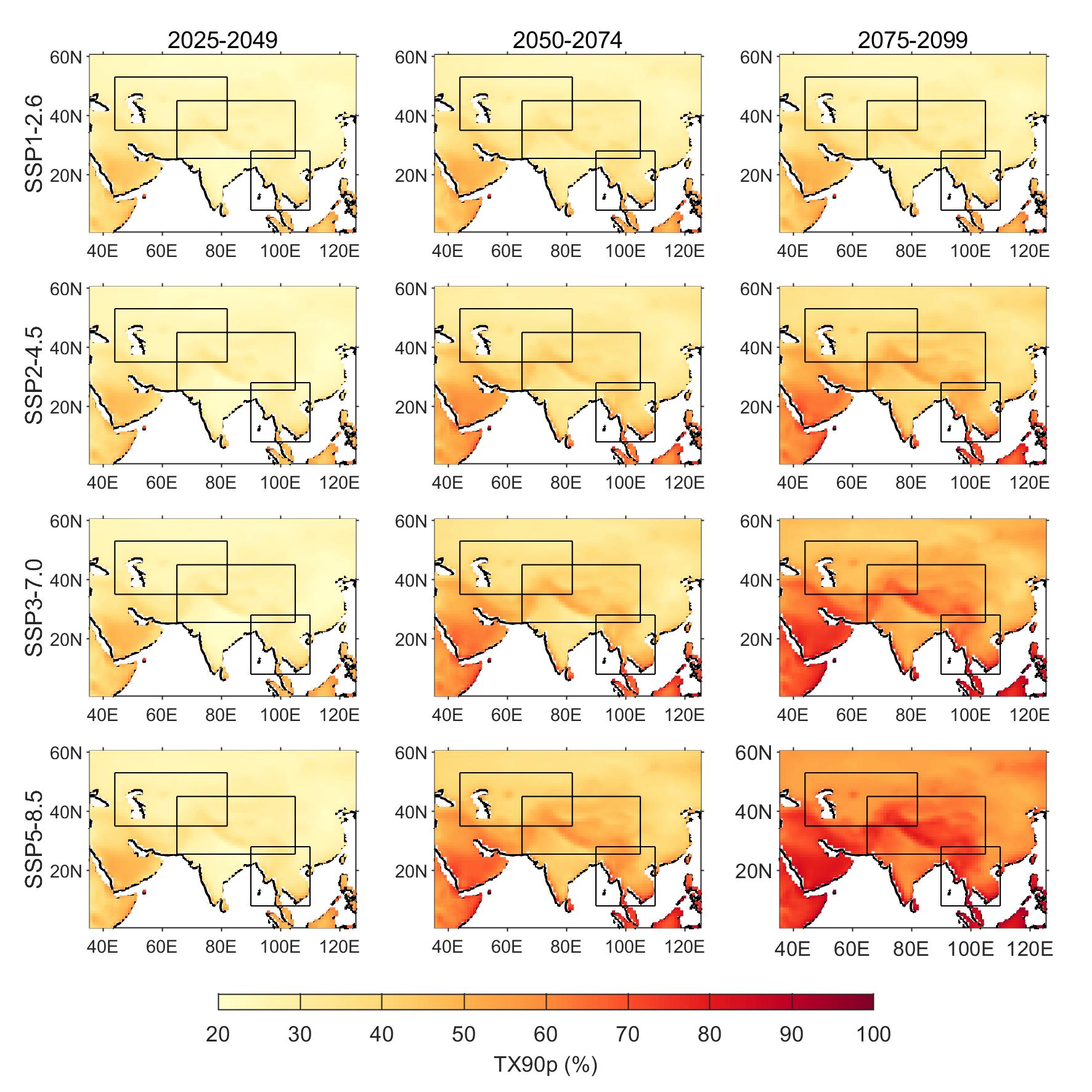


Figure S10 Same as Figure S8, but for warm days (TX90p).


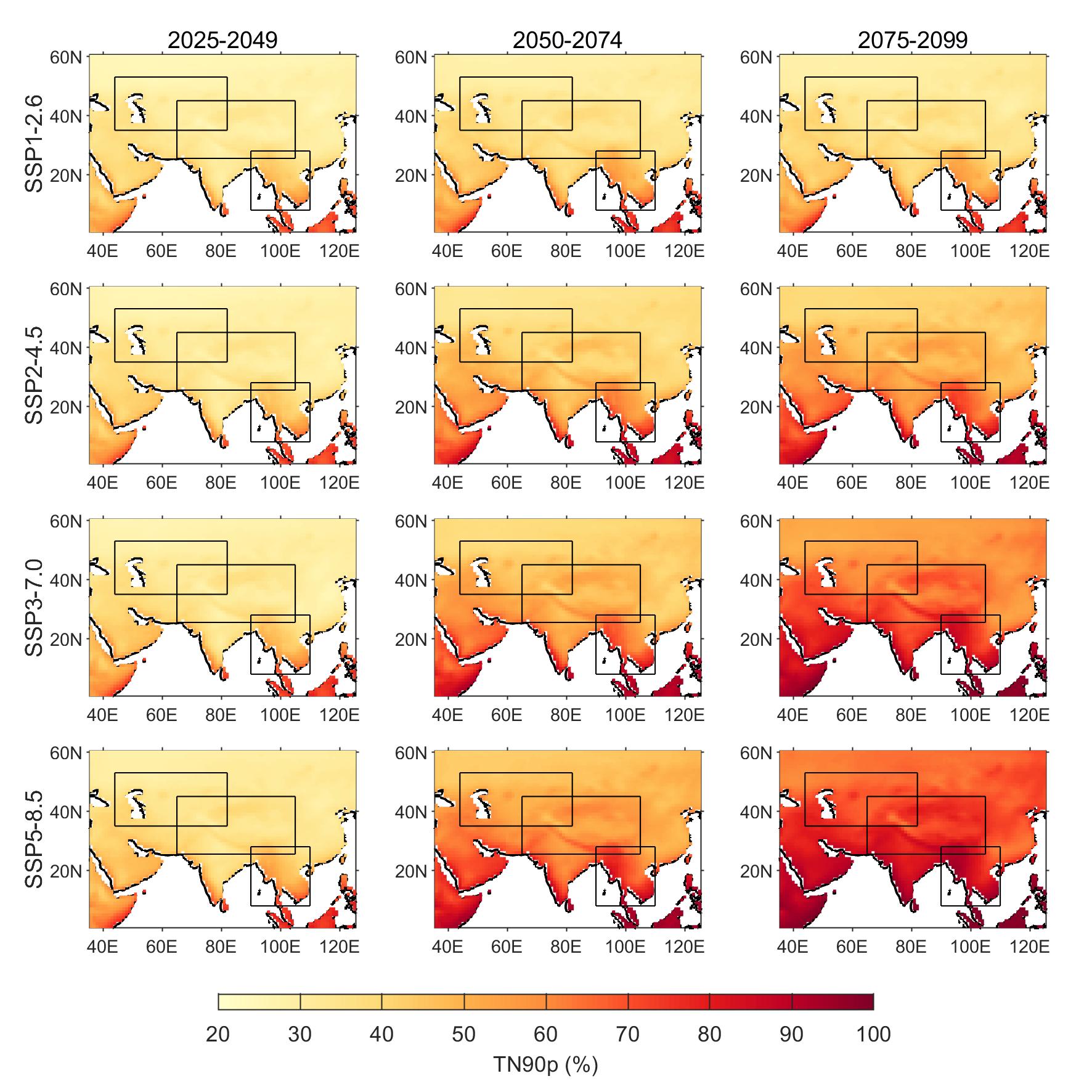


Figure S11 Same as Figure S8, but for warm nights (TN90p).
